# Association of perceived childhood socio-economic status and health with depressive symptoms among middle-aged and older adults in India: using data from LASI Wave I, 2017–2018

**DOI:** 10.1186/s12877-024-04800-0

**Published:** 2024-03-05

**Authors:** Gayatri Khanal, Y. Selvamani

**Affiliations:** School of Public Health, SRM Institute of Science and Technology (SRMIST), Kattankulathur-603 203 Chengalpattu District, Chennai, Tamil Nadu India

**Keywords:** Childhood socioeconomic status, Childhood health, Depressive symptoms, Middle-aged and older adults, LASI

## Abstract

**Background:**

Childhood adverse experience has been linked with poor health outcomes across the life course. Nevertheless, whether such an association or direction could be projected to older people’s life remains still unclear and needs to generate more evidence, particularly in India. Therefore, this study was conducted to examine the association of childhood socio-economic status and health with depressive symptoms amongst middle- aged and older adults in India.

**Methods:**

The data for the study was drawn from national representative survey “Longitudinal Ageing Study in India (LASI)” Wave I, 2017–2018 in order to conduct cross-sectional study. Multivariable regression analysis was used to examine the association of childhood socioeconomic status and health with depressive symptoms in the older population.

**Results:**

Poor childhood health was significantly and positively associated with depressive symptoms (AoR: 1.56, CI: 1.19, 2.04). Likewise, respondents who were bedridden for a month during their childhood had high odds of developing depressive symptoms (AoR: 1.16 CI: 1.01, 1.34). In addition to this, the odds of having depressive symptoms increased significantly among the average (AoR: 1.28 CI: 1.08, 1.51) and poor childhood socioeconomic status group (AoR: 1.31 CI: 1.11, 1.55) as compared to the higher socioeconomic category.

**Conclusions:**

Childhood socioeconomic status and health have a significant role in determining mental health in later life. Results suggest that considering childhood socioeconomic status and health is important while diagnosing depression in older population in order to identify the significant associated factors in early childhood and thus help in preventing depressive symptoms in later life.

## Background

An enlarge number of literatures acknowledge the relationship between adverse early-life socioeconomic status and poor mental health outcomes especially depression in later life [1–3]. Depression is one of the leading causes of disability among middle-aged and older people, affecting an estimated 322 million people worldwide which contributes in majority to the global disease burden [4, 5]. The elderly population suffering from depression has a higher risk of cognitive dysfunction, senile dementia, suicide and decreased quality of life which imposes a heavy burden on the society and family [6, 7]. According to WHO, approximately 1 in every 6 older adults aged 60 years and above suffer from a mental disorder, such as depression [8]. The number of older adults suffering from depression is increasing day by day in India with the current prevalence rate ranging from 29 to 34.4% [9, 10]. Regarding the Indian scenario, the burden of depression is soaring among adults and particularly the older age groups [9–12]. Furthermore, the escalating rate of depression in the Indian population is an emerging public health challenge that cannot be ignored [11, 12].

India, the most populated country, is experiencing a rapid demographic change characterized by the rising number of middle-aged and older people [13]. Socio-economic disparities are high in India with the significant number of people falling in lower socio-economic level with unstable financial conditions [14, 15]. Moreover, low socio-economic status and impoverished condition increase the vulnerability to mental health problems [16].

Childhood health experience is considered as a risk factor for depression [17–19]. Existing literature has highlighted that early detection of depression is inextricable from childhood experiences [20] because depression is jointly caused by biological, psychological, and social factors [21]. Furthermore, studies using life course framework clearly explained that childhood experiences will produce an enduring impact and consequences throughout the course of life [22, 23]. According to the life course epidemiology theory, social and economic exposures experienced during a particular developmental stage of life, such as childhood and adolescence, can have a significant and lasting impact on health outcomes in later life [21–23]. The changes in the mental health status of middle-aged and elderly people are especially affected by several factors [2, 24, 25] including the childhood socioeconomic status. However, how much it has impacted to the depression is yet be explored by further studies.

Association between adverse childhood experiences, unfavorable childhood socioeconomic status, poor childhood health and future mental illness have become one of the emerging fields in medical research to investigate the health of elderly people in recent years. However, whether such association or direction can be reflected in elderly life still remains unclear. In India, trivial studies have been conducted on adult depression triggered by childhood experiences depending on household economy and childhood health [16]. Hence, this study was carried out to identify an association of childhood socio-economic status and health experiences with the emergence of depressive symptoms in later life. The findings of study provide novel evidence on the perceived childhood socioeconomic and health with depression in middle and old aged people as well as shed light on importance of childhood socioeconomic and health to preventing, promoting of mental health in later life of an individuals.

## Methods

### Data and sample

This paper utilized the data from the first wave of the nationally representative survey “Longitudinal Aging Study in India” (LASI WAVE-I, 2017–2018) undertaken in close collaboration with the Harvard T. H. Chan School of Public Health and the University of Southern California, Ministry of Health and Family Welfare, Government of India, the United Nations Population Fund-India and coordinated by the International Institute for Population Sciences (IIPS), Mumbai, India. The LASI survey embraces a multistage stratified area probability cluster sampling design with a three-stage and four stage sampling design used in rural and urban areas, respectively. The first stage was the selection of sub-districts (Tehsils/Talikas), followed by the selection of villages in rural areas and wards in urban places from the selected sub-districts. In the third stage, a fixed number of households (32 from each village and 35 from each CED) were selected from the rural and urban areas. In Urban areas, first Census Enumeration Block (CED) was randomly selected in each urban ward and then households were selected from each CED. The detailed information about the survey and it’s methodology is published elsewhere [26, 27].

LASI provides information for all Indian states and union territories on demographics, household economic status, chronic health conditions, symptoms-based health conditions, functional health, mental health and other components of middle-aged and older adults in India. Ethical approval for LASI survey was obtained from various collaborative organizations and individuals, household informed consent, consent for dried sample collection and consent for proxy interview forms were filled out before starting the survey. Researchers have requested and got permission from appropriate authority (International Institute for Population Sciences, Mumbai, India) for utilization of the LASI Wave I data. After receiving the data, separate dataset was created on the basis of outcome variables (depressive symptoms). Then each desired variables were identified and extracted in the data set by using questionnaire. Data extracted from the primary data set were age, sex, place of residence, caste, marital status, education, The Monthly Per Capita Expenditure (MPCE) quintile, living status, self-rated health, presence of multimorbidity, depressive symptoms, perceived childhood socio-economic status and self–reported childhood health, Respondents aged 45 years and older were considered eligible for the study. The eligible sample size for the study was 73,396.

### Outcome variable

The outcome variable in this study was depressive symptoms measured using a shortened set of ten symptomatic questions based on the Centre for Epidemiology Studies Depression Scale (CES-D). The revised short version of the CES-D was used to assess depressive symptoms which was originally developed by Radloff [28, 29]. The tool comprises of 10 questions with a total score ranging from 0 to 10 [30, 31]. This tool includes three items on depressed affect, five items on somatic symptoms, and two items on positive affect. Response options included are “Rarely or none of the time (< 1 day)”, sometimes (1 or 2 days) were scored as zero, and “often (3 or 4 days)” “Most or all of the time (5–7 days)” were scored as 1 [30, 31], with items 5 and 8 being scored in reverse. The total score ranges from 0 to 10 and a score of four or more is considered positive for depressive symptoms. This tool has been validated in various settings including India [42].

### Predictor variables

#### Perceived childhood socio-economic status

The childhood socio-economic status was assessed by self-reported childhood family economic status questions as follows: When you were a child (from birth to 16 years), compared to other families in your community, how was your family’s financial situation? Scoring of self–reported childhood family economic status was performed on a 3-point scale, with 1 for “Poor”, 2 for “Average” and 3 for “Pretty well-off”.

#### Self–reported childhood health

Two questions in LASI were used to assess childhood health condition:

1) When you were a child (from birth to 16 years), how was your health condition? Scoring of self-reported childhood health condition was measured on a 5-point Likert scale, with 1 for “Very Good”, 2 for “Good”, 3 for “Fair”, 4 for “Poor” and 5 for “Very Poor”. We recorded very good to fair to represent good and poor and very poor to represent poor childhood health.

2) When you were growing up, before 16 years old, were you ever bedridden for a month or more because of a health problem? Response on childhood health problem was evaluated as 1 “No” and 2 “Yes”.

#### Other/control variables

The following sociodemographic and health measures were included as covariates: sex (Male and Female), age (< 50, 50–59, 60–69, 70–79 and 80 years and above), place of residence (rural and urban), caste wise: Scheduled Tribe (ST), Scheduled Caste (SC), Other Backward Class (OBC), and others. Marital status was categorized as currently married or otherwise (widowed/ divorced/separated/single). Education level or grade was recoded as 0 “No schooling”, 1 “Middle (primary) or less” and 3 “Secondary and above”. The data on Per capita household income collected and it was divided into five quintiles from the lowest to highest. Per Capita Annual Household Income refers to total household income divided by household size. It includes income from all members of the household and from all sources such as agricultural work, non-agricultural work, self-employment, remittances/gifts, wages/salaries from employment, pension income and public/private transfers (International Institute for Population Sciences (IIPS) and National Programme for Health Care of Elderly (NPHCE), MoHFW (2021). The presence of multimorbidity condition was recoded as “No disease”, “1 single disease” “2 double diseases” and 3 multiple diseases three or more than 3”.

### Statistical analysis

Descriptive statistics along with bivariate analysis was used to identify the characteristics and prevalence of depressive symptoms of the older population. Appropriate sampling weight was applied while carrying out univariate and bivariate analysis to compensate for unequal selection probabilities at various levels of selection and to compensate for non-responsiveness. Further, multivariate logistic regression analysis was performed to examine the association between childhood socio-economic status and health with depressive symptoms. The results were presented in the form of an adjusted odds ratio (AOR) with a 95% confidence interval (CI). Multicollinearity for regression model was observed using variance inflation factor (VIF). Statistical analysis was performed in STATA 15.0.

**Declarations**: This study used a secondary data set and humans were involved in this study. All the methods were carried out with relevant guidelines and regulations and ethical approval was taken from Indian Council for Medical Research (ICMR) for conducting the survey. Informed consent was obtained from all the participants prior to the interview.

## Results

### Percentage distributions are weighted

One-fourth of the eligible respondents (25.9%) were below 50 (45–50) years, 28.2% were 50–59 years, 26.8% were 60–69 years, 13.9% were 70–79 years and 5.2% were aged 80 and above. Majority of the study participants were female (58%), while 42% were males. A major proportion of the respondents (75.6%) were married while the rest were grouped as others (widowed, divorced, separated, single). Two–third (68.2%) of the respondents were residing in rural areas. Majority of the respondents were from the “OBC” category (44.9%), one-fifth were SC’s (19.6%) and around 9% were ST’s. Half of the study population (49.5%) did not go to school, 32% had secured primary or less education, and 18.5% of had completed secondary and above level of education. Income wise the proportion of eligible respondents was categorized as: poorest (21.4%), poorer (19.6%), middle (19.5%), richer (19.2%) and richest (20.3%). More than half (55.2%) of the respondents were free of multi-morbidity disease conditions, 22.8% of participants had suffered from a single chronic disease, while 12.5% and 5.5% of the study participants had suffered from two and three or more than 3 chronic conditions, respectively. Nine out of every ten mother (90.3%) were illiterate, 8.2% had passed primary or less education, and 1.5% had completed secondary and above level of education. Approximately three- fourth’s (72.4%) father of the study population never had a formal education, 19.6% had primary or less education, and only 8% had completed secondary and above level of education. Two out of five (42.1%) participants had poor SES in their childhood, while nearly half (49.8%), and 8.1% of the participants had average and pretty well-off childhood SES, respectively. Majority (87.6%) of the eligible respondents had good health during their childhood while 10.8% and 1.6% of study respondents had average and poor childhood health, respectively. Around 6% of the study participants were bedridden for a month during childhood due to the deterioration of their health condition (Table 1).

**Table 1:** Sociodemographic characteristics and the distribution of depressive symptoms among the study population, LASI Wave 1 (2017–2018), Total, *N* = 73,396

Background characteristics	N/ % Descriptive characteristics	N/% Depressive symptoms (95% CI)
**Age groups (Years)**		
< 50	25.9	23.5 (22.2, 24.7)
50–59	28.2	27.4 (25.6, 29.2)
60–69	26.8	28.8 (27.6, 30.0)
70–79	13.9	31.2 (29.4, 33.0)
80+	5.2	34.7 (31.8, 37.8)
**Sex**		
Male	42.0	25.6 (24.5, 26.7)
Female	58.0	29.1 (28.1, 30.0)
**Marital status**		
Currently married	75.6	25.2 (24.4, 26.1)
Otherwise	24.4	35.2 (33.5, 36.8)
**Place of residence**		
Rural	68.2	28.4 (27.8, 29.0)
Urban	31.8	25.9 (24.0, 28.0)
**Caste**		
Scheduled Tribe (ST)	8.8	25.4 (23.6, 27.3)
Scheduled Caste (SC)	19.6	31.3 (29.9, 32.6)
Other backward class (OBC)	44.9	27.5 (26.3, 28.7)
None of above	26.6	25.4 (24.4, 26.5)
**Education**		
No schooling	49.5	31.6 (30.7, 32.6)
Middle or less	32.0	25.6 (24.5, 26.6)
Secondary and above	18.5	20.5 (18.2, 23.0)
**Income quintile**		
Poorest	21.4	32.7 (31.3, 34.1)
Poorer	19.6	29.4 (28.1, 30.8)
Middle	19.5	27.6 (26.2, 29.0)
Richer	19.2	25.8 (24.4, 27.2)
Richest	20.3	21.9 (20.0, 24.0)
**Mothers’ education**		
No schooling	90.3	28.4 (27.7, 29.2)
Middle (primary) or less	8.2	22.0 (19.9, 24.2)
Secondary and above	1.5	15.5 (10.7, 21.9)
**Fathers’ education**		
No schooling	72.4	29.3 (28.5, 30.1)
Middle (primary) or less	19.6	24.4 (23.0, 25.9)
Secondary and above	8.0	21.7 (17.4, 26.8)
**Childhood SES**		
Pretty well off	8.1	23.6 (21.2, 26.0)
Average	49.8	27.1 (25.9, 28.3)
Poor	42.1	29.0 (28.1, 30.0)
**Ever bedridden for a month**		
No	94.0	27.4 (26.6, 28.2)
Yes	6.0	30.7 (27.1, 34.6)
**Childhood Health**		
Good	87.6	26.5 (26.5, 27.2)
Average	10.8	35.0 (31.6, 38.6)
Poor	1.6	40.7 (35.0, 47.0)
**Multimorbidity**		
No disease	55.2	25.0 (24.1, 26.0)
1 (Single) disease	26.8	28.7 (27.1, 29.9)
2 (Double) diseases	12.5	33.6 (30.1, 36.5)
3+ (Multi) diseases	5.5	35.2 (30.9, 39.8)
Total (N)	100 (73,396)	27.6 (26.8, 28.4)

The overall prevalence of depressive symptoms among eligible study participants was 27.6%. A higher prevalence of depressive symptoms was noticed among females than males (29.1% vs. 25%). The proportion of the participants having depressive symptoms inflated with increased age from 23.5% (in less than 50 years) to 34.7% (in 80 years or more) Similarly, depressive symptoms were high among the widowed/divorced/separated/ never married groups as compared to the married ones (35.2% vs. 25.6%). Respondents residing in rural areas reported higher prevalence of depressive symptoms (28.4%). The prevalence of depressive symptoms decreased with increase in wealth quintile. 32.7% of the study population with poorest quintile and 21.9% with richest quintile were depressed. The prevalence of depressive symptoms among the study population was reduced with the increased education level of father and mother (Table 1).

Furthermore, the prevalence of depressive features was higher among those who reported their health to be “poor” (43.7%). Higher prevalence was found among the participants with poor childhood health (40.7%). Similarly, the lower prevalence was noticed among the study participants who belonged to pretty well-off childhood SES (23.6%) as compared to the poor childhood SES (29%) category. Highest (35.2%) and lowest (25%) prevalence of depressive symptoms was recorded among the respondents who had suffered from multi-morbid conditions and absence of the chronic conditions, respectively (Table 1).

**Table 2:** Regression results of life course socioeconomic status, health and depressive symptoms among middle-aged and older adults in India (*N* = 73,396), LASI Wave 1

Background characteristics	COR (95% CI)	AOR (95% CI)
**Age groups (Years)**		
< 50	Reference	Reference
50–59	1.23***(1.09, 1.37)	1.14**(1.02, 1.27)
60–69	1.32***(1.20, 1.45)	1.15***(1.04, 1.28)
70–79	1.48***(1.32, 1.65)	1.17**(1.02, 1.35)
80+	1.73***(1.49, 2.01)	1.27***(1.06, 1.53)
**Sex**		
Male	Reference	Reference
Female	1.19***(1.10, 1.28)	1.05(0.96, 1.15)
**Marital status**		
Currently married	Reference	Reference
Otherwise	1.60***(1.47, 1.75)	1.33***(1.21,1.46)
**Place of residence**		
Rural	Reference	Reference
Urban	0.88**(0.79, 0.98)	0.91**(0.83,0.99)
**Caste**		
Scheduled Tribe (ST)	Reference	Reference
Scheduled Caste (SC)	1.33***(1.18, 1.50)	1.07(0.94,1.23)
Other backward class (OBC)	1.11*(0.99, 1.25)	0.93(0.82,1.06)
None of above	1.00 (0.89, 1.12)	0.94(0.81,1.08)
**Education**		
No schooling	Reference	Reference
Primary or less	0.74***(0.69, 0.79)	0.88**(0.80,0.96)
Secondary and above	0.55***(0.47, 0.64)	0.67***(0.55, 0.82)
**Income quintile**		
Poorest	Reference	Reference
Poorer	0.85***(0.78, 0.94)	0.88**(0.79, 0.98)
Middle	0.78***(0.71, 0.86)	0.88**(0.79, 0.97)
Richer	0.71***(0.64, 0.78)	0.81***(0.73, 0.90)
Richest	0.57***(0.50, 0.65)	0.68***(0.59, 0.77)
**Mother education**		
No schooling	Reference	Reference
Primary	0.71***(0.62, 0.80)	0.99(0.83, 1.17)
Middle (secondary) or above	0.46***(0.30, 0.70)	0.76(0.46, 1.24)
**Fathers’ education**		
No schooling	Reference	Reference
Primary	0.74***(0.69, 0.79)	0.98(0.88, 1.10)
Middle (secondary) or above	0.55***(0.48, 0.63)	0.89(0.73, 1.09)
**Childhood SES**		
Pretty well off	Reference	Reference
Average	1.20**(1.03, 1.39)	1.28***(1.08, 1.51)
Poor	1.32***(1.14, 1.52)	1.31***(1.11, 1.55)
**Ever bedridden for a month**		
No	Reference	Reference
Yes	1.17*(0.97, 1.40)	1.16**(1.01, 1.34)
**Childhood Health**		
Good	Reference	Reference
Average	1.49***(1.27, 1.74)	1.15*(0.99, 1.32)
Poor	1.90***(1.49, 2.43)	1.56***(1.19, 2.04)
**Multimorbidity**		
No disease	Reference	Reference
1 disease (single)	1.20***(1.11, 1.30)	1.25***(1.16, 1.36)
2 diseases (double)	1.51***(1.32, 1.74)	1.60***(1.39, 1.85)
3 + diseases (multi)	1.62***(1.33, 1.99)	1.82***(1.50,2.20)
Pseudo R^2^		0.0569

*** *p* <.001, ** *p* <.005, * *p* <.01 Results are adjusted for states

Table 2 presents the adjusted odds ratios obtained from logistic regression analysis to determine the effects of life course socioeconomic status and childhood health on depressive symptoms among middle-aged and older adults in India. Respondents who were living with spouse, family or other people have a significantly lower probability of having depressive symptoms compared to those living alone. The odds of reporting depressive symptoms significantly decreased for a unit increase in education as compared to no education for instance, primary education (AoR: 0.88 CI: 0.80, 0.96), middle and above (AoR: 0.67 CI: 0.55, 0.82). Respondents who belong to the richer (AoR: 0.81, CI: 0.73, 0.90) and richest (AoR: 0.68, CI: 0.59, 0.77) wealth categories were less likely to experience depressive symptoms compared to poorest income groups. Poor childhood health was significantly and positively associated with depressive symptoms (AoR: 1.56, CI: 1.19, 2.04). Likewise, respondents who were bedridden for a month during their childhood had high odds of having depressive symptoms (AoR: 1.16 CI: 1.01, 1.34) in reference to those who were not. In addition to this, the odds of having depressive symptoms increased significantly among the average (AoR: 1.28 CI: 1.08, 1.51) and poor childhood socioeconomic status group (AoR: 1.31 CI: 1.11, 1.55) as compared to the pretty well-off childhood socioeconomic groups. Respondents with an increasing number of multiple diseases have higher odds of experiencing depressive symptoms in comparison to those who were free of chronic diseases. The odds of depressive symptoms were higher among respondents suffering from a single disease (AoR: 1.25 CI: 1.16, 1.36), two diseases (AoR: 1.60 CI: 1.39, 1.85) and multiple diseases AoR: 1.82 CI: 1.50, 2.20). (Table 2).

## Discussion

In this study, the association of childhood socioeconomic status and health with the presence of depressive symptoms in late adulthood was assessed using a nationally representative data. The above analysis suggested a significant association between childhood socioeconomic status and health with the likelihood of depressive symptoms in later life. Furthermore, factors such as educational status, presence of multiple long-term co-morbid conditions and income were significantly associated with depressive symptoms.

The results from the present study, which demonstrated the significant association of better socioeconomic position during childhood with depressive symptoms, underpins findings from previous observational studies conducted across the globe [2, 3, 32–37]. Similarly, evidence from various meta-analysis articles on social correlates of depression had declared conclusively that the low socioeconomic status of an individual had higher odds of being depressed [25, 38]. Additionally, findings from a study reinforce our findings by stating the important relationship between lower socioeconomic status and vulnerability to mental health conditions [16]. Similarly, other previous research has also indicated that adverse childhood experiences are profoundly linked to early identification of depression [20], as depression is a result of a combination of environmental, biological and psychological [21]. Furthermore, research employing the life course framework made it abundantly evident that events experienced throughout childhood will have a lasting effect and implications for the rest of one’s life [22, 23].

According to prospective study that tracked depression for up to 15 years has further strengthened the findings of this study highlighting, childhood socioeconomic pressure, lack of social support, and physical symptoms in adulthood all have an impact on depression [39]. However, the clear nature of impact yet be explored by further extensive studies. Low childhood socioeconomic status had a long-latency effect on the onset of depression in older persons, according to a Japanese prospective cohort study [40].

This study found that poor childhood health conditions were risk factors for the emergence of depression in late life. Results from an observational study on childhood health and depression in older life by Min Yao and colleagues revealed clearly that poor childhood health conditions increase the odds of being depressed [17]. Additionally, the results of this study strengthen the life course theory that early childhood adverse experiences have a significant impact on depression in late life [18–20].

The plausible explanation behind the association of childhood socio-economic status and health with depression would be that childhood is a crucial period providing an opportunity to decrease the number of episodes of low socioeconomic status that an individual may experience throughout their life course [41]. Socio-economic disparities in India is a long-standing issue because of which people are living in impoverished conditions escalating the vulnerability to the alter mental health condition [14, 15]. Similarly, childhood socioeconomic status has also been shown to be closely related to physical trauma, emotional trauma, and/or sexual abuse during childhood [42]. Hence is important to deal with socioeconomic disparities of the community by boosting access to basic health and education services and reducing barriers to labor market participation [1].

The current study outlines that an increase in education level has a significant role in decreasing depressive symptoms which was similarly supported in a meta-analysis by Lorant and colleagues [25]. The finding that the greater number of years of education accomplished, the weaker the impact of the depression was identified in studies done by Schaan et al., and Sheikh et al. [36, 37]. Socioeconomic status and education have a significant role in determining mental health, physical health, people perception towards their health and quality of life; thereby avoiding from the adverse physical, social and environmental circumstances [2, 43, 44].

This research will add an empirical basis for the study on the childhood socioeconomic conditions and health on the depression of older adults and provide a scientific basis and intervention directed for further research. Besides this, this study also contributes to a deeper understanding of the relationship among childhood socioeconomic status, health, and depression symptoms later in life as well as strengthen the importance on life course epidemiology.

### Strengths and limitations of the study

This study was used nationally and state-representative data sets. Therefore, the results can be generalized for the national level. Validated scale used in this study to assess depressive symptoms, further strengthens our study. This study has the following limitations. First of all, this study has utilized the CES-D to assess depression in older adults which may result in an underestimation or overestimation of depressive symptoms among the study participants. Secondly, present study rely on self-reported data for childhood socioeconomic condition and childhood health experiences which may be affected by recall bias. Recall bias may hamper the appropriate measurement of the predictive variables in this study. Thirdly, subjective questionnaire was used to measure childhood socioeconomic status and health condition, instead of objective tool, because researches indicates that subjective childhood socioeconomic status and health has a stronger correlation with mental health outcomes than objective [45]. Further, the data used in this study was based on cross-sectional study. A longitudinal data that could explore the patterns of change and the dynamics of the individual behavior and health [46], would have provided a more precise finding.

## Conclusions

In conclusion, this study showed that childhood socioeconomic status and health have a profound role in the emergence of depressive symptoms in older adults. Factors like educational level, poor self-rated health, multimorbidity and income were positively associated with the presence of depressive features. Results suggested that socioeconomic status and health across the early life course was detrimental in modeling the mental health in later life of the older population. Further, the results also support that the measures to improve the childhood health will automatically reflect in future life course for better mental health.

## Data Availability

This study uses secondary data which is publicly available on request to IIPS, Mumbai through https://www.iipsindia.ac.in/content/LASI-data.

## Abbreviations

AOR: Adjusted Odds Ratio
COR: Crude Odds Ratio
CI: Confidence Interval
(CES-D): Center for Epidemiology Studies Depression Scale
CED: Census Enumeration Block
IIPS: International Institute for Population Sciences
CSES: Childhood Socio-economic Status
SRH: Self-rated Health
LASI: Longitudinal Aging Study in India
MPCE: Monthly Per Capita Expenditure
